# Investigation of class-F power amplifier in the presence of the second and fourth harmonics of input voltage

**DOI:** 10.1038/s41598-024-58494-w

**Published:** 2024-04-02

**Authors:** Parastoo Rostami, Akram Sheikhi

**Affiliations:** Engineering Department, Faculty of Engineering, Lorestan University, Khorramabad, 68151-44316 Iran

**Keywords:** Engineering, Electrical and electronic engineering

## Abstract

In this paper, a thorough theoretical investigation of high-efficiency class-F power amplifier (PA) is undertaken to drive, considering the second and fourth harmonics of input voltage. The precise analytical expressions of the gate-source voltage, drain current, output power, and efficiency are extracted. Maximum normalized output power and maximum drain efficiency from the initial phase and amplitude of the second and fourth harmonics of input gate-voltage can be achieved. The simulation of class-F PA using a GaN CGH40010F transistor has been done to validate the theoretical analysis. Addition of the fourth harmonic gate voltage along with the second harmonic improves the output power and efficiency of the PA by 0.7 dBm and 4.1%. Based on the simulation with realistic elements, a highly efficient PA operating from 1.8 to 2.2 GHz is implemented. The fabricated PA provides an efficiency of 72–87.6%, PAE of 65–80% and an output power of 39.3–41 dBm.

## Introduction

The rapid growth of the wireless communication industry has led to increased demands on the radio frequency RF transmission systems. Power amplifiers (PAs), being one of the most energy-consuming components in the RF transmission systems and significantly impact its performance. In modern wireless communication systems, PAs play a critical role and contribute to the power consumption of the whole system. Consequently, there is a strong focus among researchers on designing high-efficiency PAs Ref.^1–16^. In Ref.^1^, a theory is presented for continuous class-F harmonic tuned PAs biased above pinch-off. This theory, applied to a class-F harmonic tuned PA under class-AB bias conditions, identifies a shifted fundamental impedance space and expanded second harmonic impedance space with varying conduction angles. The implemented PA showcases impressive performance, achieving a PAE surpassing 70% across a 400 MHz bandwidth. A high-efficiency inverse class-F PA is presented in Ref.^2^. This paper addresses efficiency degradation in PAs through waveform engineering. It introduces a novel method for estimating theoretical performance at minimum efficiency, utilizing an active load-pull (ALP) system. In Ref.^3^ a 5 GHz Class-J PA featuring an output matching network with a lumped $\pi$-type configuration has been introduced. The Class-J PA is designed to elevate performance metrics, delivering outstanding results with a peak output power reaching 27 dBm, a maximum power gain of 13.7 dB, and a small-signal gain of 17 dB. Notably, it accomplishes these feats within a bandwidth of approximately 500 MHz. While the efficiency and gain of this Class-J amplifier are high, its bandwidth is not significantly greater compared to other PAs. In Ref.^4^, the analysis of high-efficiency class-F and inverse class-F PAs has been presented. The study focuses on the examination of crucial parameters, such as nonlinear capacitance and knee voltage, and their impact on efficiency. A reconfigurable dual-band class-F PA design employing stub switching, that utilizes a GaN-HEMT is introduced in Ref.^5^. By incorporating a harmonic tuning structure, a highly efficient dual-band PA with fewer components and reduced size is proposed. In Ref.^6^ an inverse class-F PA utilizing a microstrip multimode bandpass impedance transformer (BPIT) is proposed. By employing a modified quad-mode BPIT operation, the design achieves a flat wideband response with a small size. A design for an asymmetric Doherty PA (DPA) composed of a class-F and an inverse class-F carrier amplifier has been introduced in Ref.^7^. By considering various conditions for load-harmonics, the DPA generates an uneven current waveform. This asymmetry leads to an improved fundamental current and also performance enhancement at peak output power. In Ref.^8^, a new concept is introduced for a harmonic control circuit in class-F PA, utilizing the potential of a bowtie-shaped harmonic control circuit. The load network effectively achieves the desired load resistance at the $f_0$, $2f_0$ and $3f_0$. Manipulating the input voltage harmonics of a PA improves its performance. An inverse class-F PA incorporating a power combiner and a multi-harmonic resonance filter has been introduced in Ref.^9^. In Ref.^10^, the effectiveness of the class-F/E has been proven through lumped elements and transmission lines design examples. In Ref.^11^, the approach builds upon the inverse class-F specifications and expands it to a range of requirements that enable superior performance in terms of efficiency and output power compared to Class-B PAs. In Ref.^12–16^, manipulating the input gate-voltage by adding a second harmonic is affected the performance of PA, that results in an improvement in output power and efficiency. In Ref.^12^, a research is conducted on the class-F PAs by introducing a second-harmonic of input gate-source voltage to the gate of the transistor. The analysis in Ref.^13^ aims to investigate the effects of input second-harmonic nonlinearity on the accomplishment of the continuous wideband class-F PAs. In Ref.^14^, a continuous inverse Class-GF PA has been introduced based on an analytical formula for the drain current to manipulate the second harmonic impedance and expand the design possibilities of the output matching network resistive. In Ref.^15^, a high-efficiency inverse class-F PA is designed, by an emphasis on the input second harmonic effect for efficiency enhancement. A comprehensive analysis of a wideband inverse class-F PA in the presence of input-output nonlinearity is presented in Ref.^16^. As can be seen, other works till now just investigated the effect of second-harmonic of input gate-voltage on the PAs performance. In this work, the impact of the fourth harmonic input gate-voltage in addition to the second harmonic will be investigated. The comprehensive expressions of the drain current, output power and drain efficiency of class-F PA considering two harmonics of input gate-voltage to the gate node are derived. The paper is categorized stated as bellow: In “class-F PA considering second and fourth harmonics of input gate-voltage” section, the impact of the second and fourth harmonics of the input gate-voltage on class-F PA performance will be discussed. To verify the theory, the simulation results of class-F PA will be presented in “Simulation and implementation results” section. The last section also includes the conclusion.

**Figure 1:**
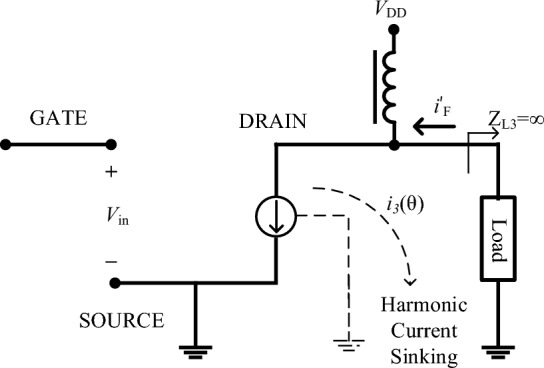
The schematic of the conventional class-F PA.

## Class-F PA considering second and fourth harmonics of input gate-voltage

The configuration of a conventional class-F PA is illustrated in Fig. 1. The drain current of the class-F can be represented:

1 $$\begin{aligned} i_F(\theta )=\frac{I_{max}}{\pi }\times {[1+\frac{\pi }{2}\sin (\theta )-(\frac{2}{3}\cos (2\theta )+\frac{2}{15}\cos (4\theta ))]} \end{aligned}$$

where $I_{max}$ is the maximum drain current. Also, the gate-source voltage can be written as a sinusoidal voltage equation:

2 $$\begin{aligned} V_{gs}=V_{gs0}+V_{gs1}\cos \theta \end{aligned}$$

where $V_{gs0}$ and $V_{gs1}$ are DC and fundamental components of the gate voltage. We can presume that drain current is calculated by nonlinearity products of gate-source voltage as follows:

3 $$\begin{aligned} i_F(\theta )=a_{0}+a_{1}{V_{gs(\theta )}+a_{2}{V_{gs}^2(\theta )}+a_{3}{V_{gs}^3(\theta )}+a_{4}{V_{gs}^4(\theta )}+a_{5}{V_{gs}^5(\theta )}} \end{aligned}$$

where $a_{i}$ are the gate voltage coefficients. Applying (3) to (2), the drain current can be written as:

4 $$\begin{aligned} i_F(\theta )=I_{dc}+i_{1}(\theta )+i_{2}(\theta )+i_{3}(\theta )+i_{4}(\theta )+i_{5}(\theta ) \end{aligned}$$

Class-F PAs deliver no third harmonic current. Thus, their load currents can be explained as:

5 $$\begin{aligned} { i^{\prime }}_F(\theta )=i_F(\theta )-i_{3}(\theta ) \end{aligned}$$

where the load current of the third harmonic frequency $i_{3}(\theta )$ is:

6 $$\begin{aligned} i_{3}(\theta )=r_{3}\cos (3{\theta })+q_{3}\sin (3{\theta }) \end{aligned}$$

where $r_{3}$ is the real part and $q_{3}$ is the imaginary part of the $i_{3}(\theta )$. Since the class-F load current does not contain the third harmonic, so we have:

7 $$\begin{aligned} {\frac{1}{2\pi }}{\int _{-\pi }^{\pi }i_F^{\prime }\cos (3\theta )d\theta }=0 \end{aligned}$$

8 $$\begin{aligned} {\frac{1}{2\pi }}{\int _{-\pi }^{\pi }i_F^{\prime }\sin (3\theta )d\theta }=0 \end{aligned}$$

Solving the Fourier expansions equations, the coefficients of the harmonic currents can be calculated as:

9 $$\begin{aligned} r_{3}= & {} 0 \end{aligned}$$

10 $$\begin{aligned} q_{3}= & {} {V^3_{gs1}}(\frac{1}{4}a_{3}+a_{4}V_{gs0}+\frac{5}{2}a_{5}{V^2_{gs0}}+\frac{5}{16}{V^2_{gs1}}) \end{aligned}$$

The harmonic coefficients of the $i_{5}(\theta )$ can be calculated as:

11 $$\begin{aligned} r_{5}= & {} 0 \end{aligned}$$

12 $$\begin{aligned} q_{5}= & {} \frac{a_{5}V^5_{gs1}}{16} \end{aligned}$$

**Figure 2:**
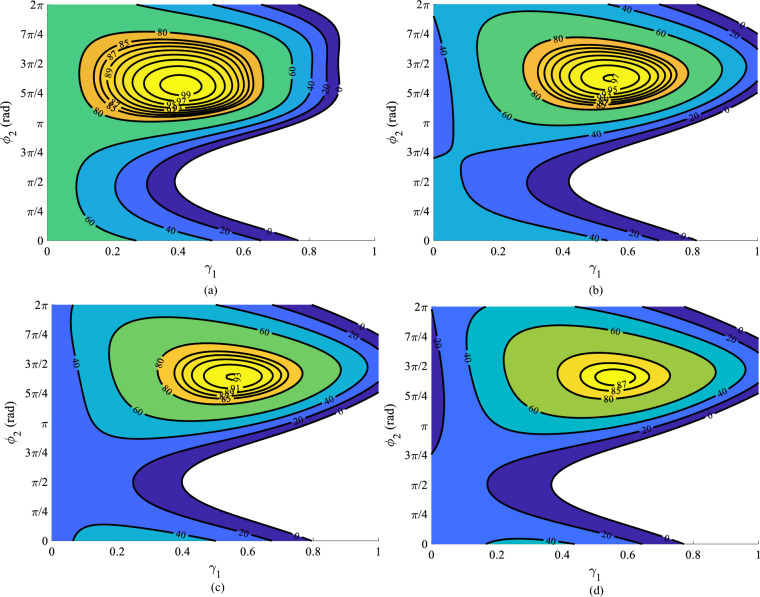
The drain efficiency of class-F PA in terms of the $\gamma _1$ and initial phase $\phi _2$ when (**a**) $\gamma _{2}=0.1$, (**b**) $\gamma _{2}=0.45$, (**c**) $\gamma _{2}=0.5$, and (**d**) $\gamma _{2}=0.55$.

The second and fourth harmonics of input gate-voltage with phase $\phi _2$, $\phi _4$ and an initial amplitude of $V_{gs2}$, $V_{gs4}$ is added to the input gate-voltage of the transistor. Therefore, the gate-source voltage is reconstructed:

13 $$\begin{aligned} V_{gs}=V_{gs0}+V_{gs1}\cos \theta +V_{gs2}\cos (2\theta +\phi _{2})+V_{gs4}\cos (4\theta +\phi _{4}) \end{aligned}$$

as a result:

14 $$\begin{aligned} i_{F_{2nd, 4th}}(\theta )=I_{dc}+i_{1}(\theta )+i_{2}(\theta )+i_{3}(\theta )+i_{4}(\theta )+i_{5}(\theta )+...+i_{15}(\theta ) \end{aligned}$$

Consequently, the drain-source voltage can be written as:

15 $$\begin{aligned} V_{ds}=V_{dc}-\alpha [{\sin (\theta )+{M_{1}}{\sin (3\theta )}+{M_{2}}{\sin (5\theta )}}] \end{aligned}$$

where $$\alpha =\frac{4(V_{dc}-V_{k})}{\pi }$$, $$M_{1}=\frac{1}{3}$$, and $$M_{2}=\frac{1}{5}$$.

**Figure 3:**
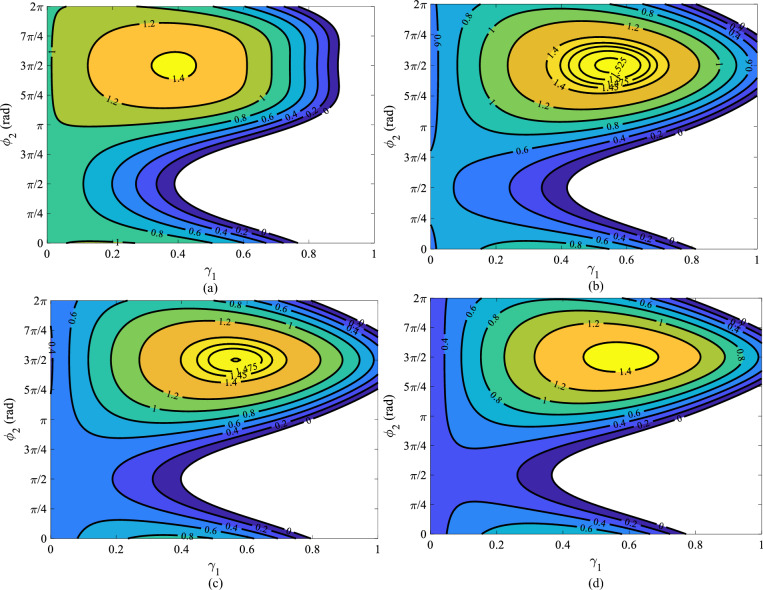
The normalized output power of class-F PA in terms of the $\gamma _1$ and initial phase $\phi _2$ when (**a**) $\gamma _{2}=0.1$, (**b**) $\gamma _{2}=0.45$, (**c**) $\gamma _{2}=0.5$, and (**d**) $\gamma _{2}=0.55$.

The normalized output power ($P_{F,norm}$), which is the proportion between the output power with second and fourth harmonics of input voltage and the output power with sinusoidal input voltage (without harmonics), is derived.

16 $$\begin{aligned} P_{F,norm}=\frac{P_{out(2nd,4th)}}{P_{out(sin)}}=\frac{V_{ds(2nd,4th)}i_{F(2nd,4th)}}{V_{ds(sin)}{i_{F(sin)}}} \end{aligned}$$

Equations (4–6) and (9–12) when incorporated into equation (1) reveal the coefficients of the drain current for the class-F PA under the influence of second and fourth harmonics of the input voltage. The class-F PA’s drain-source voltage can be determined using Eq. (15). The normalized output power of class-F PA can be calculated as:

17 $$\begin{aligned} {P_{F,norm}=}{\qquad {\frac{(A_{1}+A_{2})({\gamma _{1}}^2+{\gamma _{2}}^2)+A_{3}({\gamma _{1}}^2{\gamma _{2}}-{\gamma _{2}}{\gamma _{1}}^2)+A_{4}+A_{5}}{A_{4}}}} \end{aligned}$$

where ($A_{1}$–$A_{5}$) are the coefficients that depends on the DC component of input voltage $V_{gs0}$, fundamental component of input voltage $V_{gs1}$, $\gamma _{1}$= $V_{gs2}$/$V_{gs1}$, $\gamma _{2}$= $V_{gs4}$/$V_{gs1}$, and ($a_{0}$–$a_{5}$). ($A_{1}$–$A_{5}$) coefficients are extracted in Eqs. (18–22).

18 $$\begin{aligned} A_{1}= & {} \frac{15}{8}a_{5}{V^5_{gs1}} \end{aligned}$$

19 $$\begin{aligned} A_{2}= & {} \frac{3}{2}a_{3}{V^3_{gs1}}+15a_{5}{V^2_{gs0}}{V^3_{gs1}}+6a_{4}{V_{gs0}}{V^3_{gs1}} \end{aligned}$$

20 $$\begin{aligned} A_{3}= & {} 3a_{4}{V^4_{gs1}}\sin (\phi _{2})+15a_{5}{V_{gs0}}{V^4_{gs1}}\sin (\phi _{2}) \end{aligned}$$

21 $$\begin{aligned} A_{4}= & {} a_{1}V_{gs1}+2a_{2}V_{gs0}V_{gs1}+3a_{3}V^2_{gs0}V_{gs1}+\frac{3}{4}a_{3}V^3_{gs1}+4a_{4}V^3_{gs0}V_{gs1}\nonumber \\{} & {} +3a_{4}V_{gs0}V^3_{gs1}+\frac{5}{8}a_{5}V^5_{gs1}+5a_{5}V^4_{gs0}V_{gs1} \end{aligned}$$

22 $$\begin{aligned} A_{5}= & {} A_{1}\times {{[-2{\gamma _{2}}^3{\gamma _{1}}+4{\gamma _{1}}^2{\gamma _{2}}^2-{\frac{8}{3}}{\gamma _{1}}{\gamma _{2}}+\frac{125}{3}{\gamma _{1}}^{2}}+{\gamma _{1}}^{2}10\sin ^2\phi _{2}}\nonumber \\{} & {} {{-\frac{16}{3}{\gamma _{1}}^3{\gamma _{2}}(\cos {\phi _{2}}+2\sin ^2{\phi _{2}}+3\sin ^2{\phi _{2}}\cos ^2{\phi _{2}})]}} \end{aligned}$$

Likewise, the efficiency considering two harmonics of input voltage can be computed as:

23 $$\begin{aligned} \eta _{F(2nd,4th)}=\frac{P_{out(2nd,4th)}}{P_{in}}\times 100 (\%)=\frac{4}{\pi }(\frac{V_{dc}-V_{k}}{V_{dc}})\frac{P_{F,norm}}{I_{dc,norm}}{\times 100} (\%) \end{aligned}$$

where $I_{dc,norm}$ is the proportion between the DC current with second and fourth harmonics of input voltage and the DC current with sinusoidal input voltage (without harmonics).

Figure 2a–d shows that the efficiency of class-F PA in the presence of second and fourth harmonics of input voltage can be better than 99% for $\gamma _{2}=0.1$. Figure 3a–d shows the normalized output power in terms of $\gamma _{1}$ and initial phase $\phi _{2}$ for various amounts of $\gamma _{2}$. It is apparent that, for $\gamma _{2}=0.1$, 0.45, 0.5, and 0.55 the normalized output power higher than 1.5 can be obtained. When $\gamma _{1}=0.57$, $\phi _{2}=11{\pi }/8$, and $\gamma _{2}=0.45$, the normalized output power 1.4 and efficiency 97% is obtained. Addition the second and fourth harmonics of input voltage to the gate node of PA, can improve class-F PA performance.

**Figure 4:**
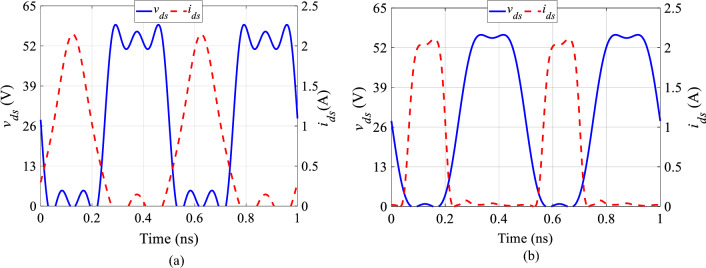
Voltage and current waveforms of class-F PA (**a**) without harmonics of input voltage, (**b**) with second and fourth harmonics of input voltage.

Figure 4a illustrates the drain voltage and current waveforms without harmonics of input voltage. Figure 4b shows the current and voltage waveforms with second and fourth harmonics of input voltage based on analysis. When we compare these waveforms to the ideal class-F voltage and current waveforms, it is evident that the theoretical results closely resemble the ideal shapes.

## Simulation and implementation results

**Figure 5:**
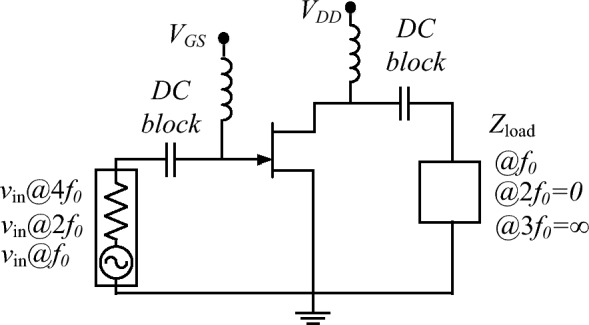
The simulation setup of class-F PA with second and fourth harmonics input gate voltage.

To further validate the analysis, the class-F PA is simulated. The setup for simulation class-F PA with input voltage harmonics in an ideal condition is demonstrated in Fig. 5. at $-2.7$ V gate voltage and 28*V* drain voltage.

The class-F PA using the GaN HEMT CGH40010F model has been simulated in Advanced Design System (ADS). The used transistor is biased The knee voltage and the maximum drain current of the transistor are 2.4V and 1A. To achieve class-F condition the second load impedance harmonic is shorted and the third impedance harmonic at the load is set to open-circuit. Values of ($a_{0}$–$a_{5}$) parameters are derived as: $a_{0}=0.7219$; $a_{1}=0.3721$; $a_{2}=0.0802$; $a_{3}=-0.0027$; $a_{4}=-0.0025$; $a_{5}=-0.0002$.

**Figure 6:**
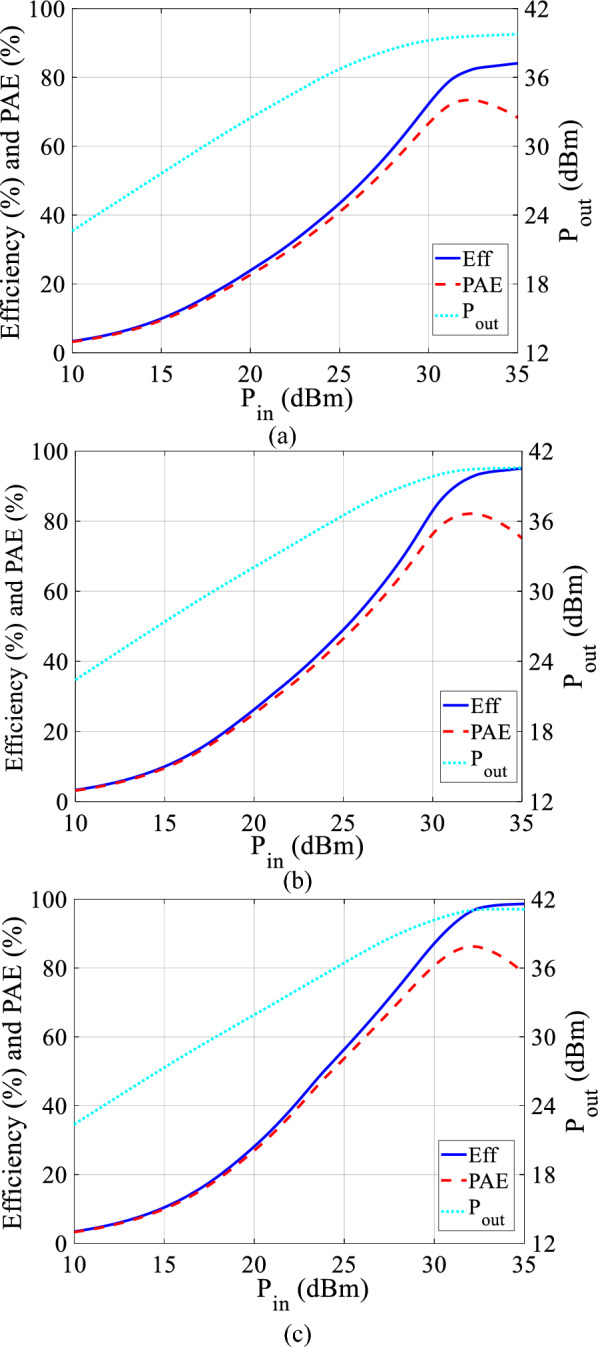
The simulated efficiency, output power, and PAE in terms of input power when $\gamma _{1}=0.57$, and $\gamma _{2}=0.45$ for (**a**) sinusoid input voltage, (**b**) second harmonic input voltage, and (**c**) second and fourth harmonic input voltage.

In Fig. 6a–c, the simulated output power, PAE and drain efficiency when $\gamma _{1}$=0.57, and $\gamma _{2}=0.45$ are shown. As is evident, the outcomes from the simulation are similar to the results in Figs. 2 and 3. According to Fig. 6a, for sinusoid input voltage without harmonics, the drain efficiency is 81.6%, PAE 73.5%, and the output power of 39.5 dBm is obtained. The introduction of the second harmonic input voltage results in substantial improvements in amplifier performance, evident in Fig. 6b. The drain efficiency experiences a notable 10.8% increase, PAE improves by 8.6%, and the output power by 0.9 dBm is increased. Furthermore, when both the second and fourth input harmonic voltages are incorporated, Fig. 6c demonstrates additional enhancements. In this case, the drain efficiency increases by 4.1%, PAE improves by 3.9%, and the output power gains an additional 0.7 dBm compared to the scenario with only the second harmonic input gate-voltage. This signifies the cumulative impact of multiple harmonic inputs on the overall efficiency and power output of the amplifier. The performance of class-F PA under various input gate-voltage in ideal situation is demonstrated in Table 1.

**Table 1:** The performances of class-F PA in different input harmonic manipulation in ideal condition.

Input voltage	Eff (%)	Pout (dBm)	PAE (%)
Without harmonic	81.6	39.5	73.5
With 2nd harmonic	92.4	40.4	82.1
With 2nd and 4th harmonics	96.5	41.1	86

In order to perform a detailed examination of class-F PA with second and fourth harmonics of input voltage under real condition, a PA with actual components is designed. The proposed class-F PA is designed based on load-pull results. Load-pull analysis helps optimize the output stage of a power amplifier by determining the load impedance that maximizes power efficiency and output power.

**Figure 7:**
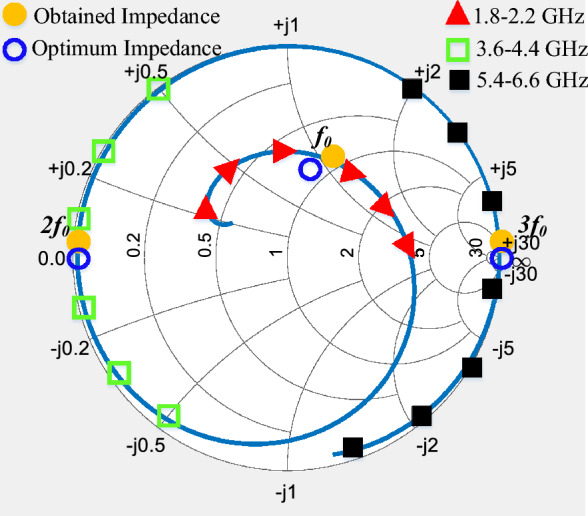
Intrinsic impedance trajectories for fundamental and harmonics of proposed PA.

Intrinsic impedance trajectories for fundamental and harmonics in the frequency range of proposed class-F PA and the obtained and optimum impedance points of fundamental, second and fourth harmonics is shown in Fig. 7. According to Fig. 7, the designed and optimum impedance points closely coincide. The design incorporates realistic components and parameters, which implies that it takes into account real-world conditions and factors in its operation.

**Figure 8:**
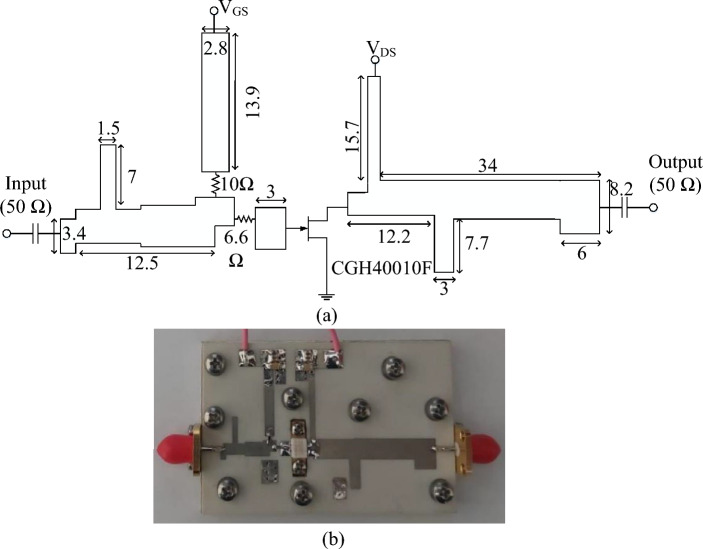
(**a**) Schematic of the designed PA (**b**) Photograph of the implemented PA. (all dimensions are in mm).

Figure 8a,b show the schematic diagram and a photograph of the fabricated class-F PA, respectively.

**Figure 9:**
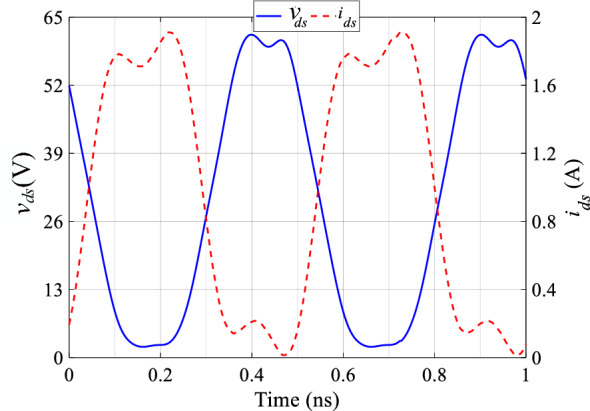
The drain voltage and current waveforms of the proposed Class F PA.

The measurement setup for characterizing the PA includes the following equipment: A vector signal analyzer 89600, a signal generator MXG N5182A by Agilent Technologies, and an infinitum MS09404A Signal Oscilloscope. The PA is implemented on a Rogers 4003C substrate with a dielectric constant ($\epsilon$) of 3.55. The class-F PA is unconditionally stabilized using two series resistors, R1 = 6.6$\Omega$ and R2 = 10$\Omega$. The input matching networks of the PA are designed to transform the source impedance to an optimal 50$\Omega$ impedance. This is important to maximize power transfer and ensure efficient operation. Impedance transformation for optimal matching is achieved using specific components. Two types of components are ATC700A series NPO Porcelain and Ceramic Multilayered Capacitors Resistors. Figure 9 illustrate the drain voltage and current waveforms of proposed class-F PA. As can be seen, the simulated results closely match the theoretically predicted class-F voltage and current waveforms.

**Figure 10:**
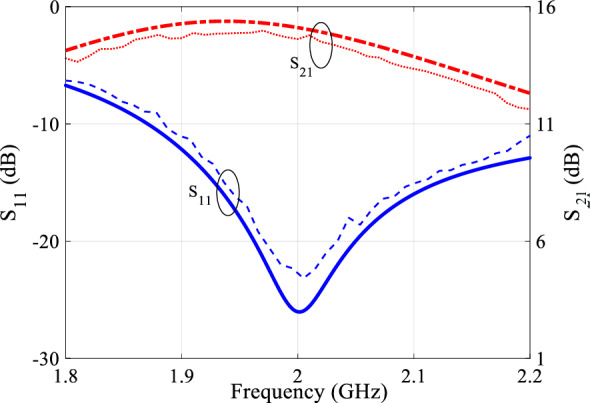
The simulated (thick lines) and measured (thin lines) S-parameters results of the proposed class F PA.

The scattering parameters of the class-F PA are depicted in Fig. 10. The PA operates within the 1.8–2.2 GHz. The average small-signal gain of the PA within this frequency range measures approximately better than 11.6 dB. The input reflection coefficient is less than − 8 dB, indicating that the PA provides good impedance matching at the input over the specified frequency range.

**Figure 11:**
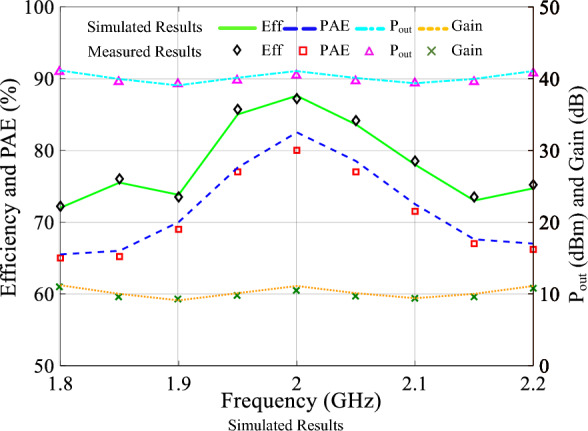
The output power, gain, PAE and efficiency of the Class F PA versus frequency.

Figure 11 presents both simulated and measured performance characteristics of the class-F PA for frequency range of 1.8–2.2 GHz. The class-F PA delivers an output power ranging from 39.3 to 41 dBm. This indicates a significant amplification of the input signal.The measured gain is obtained in the range of 9.3–11 dB. Power Utilization Factor (PUF) denotes a measure of optimal utilization of a device’s capabilities, particularly in relation to its performance in class mode. The measured PUF for the proposed class-F PA falls within the range of 0.98 to 1.02. The measured PAE of proposed amplifier is in the range from 65 to 80%. The value of efficiency falls within the range of 72 to 87.6%. The PA’s performance characteristics are reported for a frequency range of 1.8–2.2 GHz, indicating its capability to operate effectively within this frequency band.

**Figure 12:**
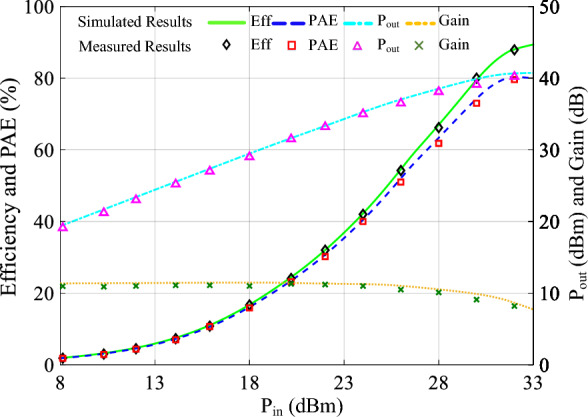
The output power, gain, PAE and efficiency of the Class F PA versus input power.

At a center frequency of 2 GHz, the performance of PA is highlighted. This is a specific point within the frequency range that is often used as a reference for performance evaluation. The performance of a class-F PA at a frequency of 2 GHz is shown in Fig. 12. The efficiency and PAE of the class-F PA at the center frequency of 2 GHz is approximately 87.6% and 80%. Also, the measured gain and the output power of the implemented class-F PA at 2 GHz is obtained as 9.5 dB and 40.5 dBm.

**Table 2:** Performance comparision with previous GaN class-F PAs.

References	Tech	*f* (GHz)	$P_{out}$ (dBm)	PUF	Gain (dB)	DE (%)	PAE (%)
^16^	GaN	1.75–2.3	53.4–54	0.99–1	14.5–15.9	65–70.7	–
^17^	GaN	2.5–2.78	37.16–39.4	0.92–0.98	10–12.36	–	50–76.4
^18^	GaN	1.88	41.06	1.02	–	75.8	70.7
^19^	GaN	1.9	44	1.05	13	73	70
^20^	GaN	2.4	40.8	1.02	–	70.9	–
This work	GaN	1.8–2.2	39.3–41	0.98–1.02	9.3–11	72–87.6	65–80

The performance of the class-F GaN PA is notably enhanced by incorporating second and fourth harmonic injections into the input voltage, as compared to prior class-F works utilizing GaN technology mentioned in Table 2. The utilization of input harmonic control leads to an improvement of a few percent points, boosting the efficiency of PA, as clearly demonstrated in Figs. 11 and 12.

## Conclusion

In this paper, an investigation of a high-efficiency class-F PA considering the second and fourth harmonics input gate-voltage of the transistor has been studied. It provides closed-form formulas for output power, drain efficiency, and drain current. The proposed analysis offers an understanding of efficiency and output power enhancement when second and fourth harmonics exist in the gate of the transistor. When the fourth harmonic of input voltage is applied, there is a noticeable improvement in the amplifier’s performance. To validate the analytical results, a class-F PA is simulated using an actual transistor. The designed and simulated PA with ideal elements achieves 96.5% efficiency, which aligns well with the theory. To further verify the theroritical analysis, the proposed PA is implemented using GaN CGH40010F technology and acheives 87.6% efficiency, 41 dBm output power and 80% PAE.

## Data Availability

The datasets used and analysed during the current study available from the corresponding author on reasonable request.
